# Lack of transparency in reporting narrative synthesis of quantitative data: a methodological assessment of systematic reviews

**DOI:** 10.1016/j.jclinepi.2018.08.019

**Published:** 2019-01

**Authors:** Mhairi Campbell, Srinivasa Vittal Katikireddi, Amanda Sowden, Hilary Thomson

**Affiliations:** a MRC/CSO Social and Public Health Sciences Unit, University of Glasgow, 200 Renfield Street, Glasgow G2 3AX, UK; b Centre for Reviews and Dissemination, University of York, York YO10 5DD, UK

**Keywords:** Systematic review, Meta-research, Methodology, Narrative synthesis, Evidence synthesis

## Abstract

**Objective:**

To assess the adequacy of reporting and conduct of narrative synthesis of quantitative data (NS) in reviews evaluating the effectiveness of public health interventions.

**Study Design and Setting:**

A retrospective comparison of a 20% (*n* = 474/2,372) random sample of public health systematic reviews from the McMaster Health Evidence database (January 2010–October 2015) to establish the proportion of reviews using NS. From those reviews using NS, 30% (*n* = 75/251) were randomly selected and data were extracted for detailed assessment of: reporting NS methods, management and investigation of heterogeneity, transparency of data presentation, and assessment of robustness of the synthesis.

**Results:**

Most reviews used NS (56%, *n* = 251/446); meta-analysis was the primary method of synthesis for 44%. In the detailed assessment of NS, 95% (*n* = 71/75) did not describe NS methods; 43% (*n* = 32) did not provide transparent links between the synthesis data and the synthesis reported in the text; of 14 reviews that identified heterogeneity in direction of effect, only one investigated the heterogeneity; and 36% (*n* = 27) did not reflect on limitations of the synthesis.

**Conclusion:**

NS methods are rarely reported in systematic reviews of public health interventions and many NS reviews lack transparency in how the data are presented and the conclusions are reached. This threatens the validity of much of the evidence synthesis used to support public health. Improved guidance on reporting and conduct of NS will contribute to improved utility of NS systematic reviews.

**What is new?**

**Key findings**
• Based on a sample of public health reviews, it is apparent that, despite being commonly used, narrative synthesis often lacks transparency.
• Synthesis methods are rarely reported, and presentation of data in the review often does not facilitate clear links between visual presentation of the data and the text.

**What this adds to what was known?**
• This is the first study to assess the adequacy of reporting of narrative synthesis of quantitative data in systematic reviews.

**What is the implication and what should change now?**
• Substantial improvements in clarity of reporting of narrative synthesis are required. There is a need for existing guidance to inform the development of a clear and concise reporting guideline for narrative synthesis.
• Greater transparency when reporting narrative synthesis will allow end users including practitioners and policy decision-makers to have greater confidence in the results of systematic reviews that use narrative synthesis.

## 1 Introduction

Well-conducted systematic reviews have an important role in supporting evidence-informed policy and practice [1], [2]. The value of systematic reviews in supporting decision-making, compared with other types of review, is their use of a transparent method to draw conclusions based on the best available evidence. While meta-analysis is a cornerstone of many systematic reviews, statistical pooling may not always be appropriate or feasible due to high levels of heterogeneity or lack of available data to calculate standardized effect estimates (e.g., standardized mean difference, odds ratio, risk ratio). Heterogeneity, both statistical and methodological, is a common issue for public health reviews where it is typical to include diverse study designs, outcomes, contexts, populations, and interventions [3]. When meta-analysis is inappropriate or not possible, data may be synthesized narratively; this method is relied on heavily by those conducting reviews addressing public health issues. For example, 74% of National Institute for Health and Care Excellence public health appraisals included NS [4].

Concerns have been raised that narrative synthesis of quantitative data (NS) lacks transparency and has substantial potential for bias [5], [6], [7]. Specifically, there is concern that conclusions of NS are based on subjective interpretation [5], [7] with a risk of over emphasizing selected results without clear justification. This lack of transparency limits assessment of the level and sources of bias in NS [5], threatens the replicability of the method, and may ultimately threaten the validity and value of review findings based on NS. However, empirical evaluations of the reporting and adequacy of NS are lacking. This article presents the findings of a systematic review that aimed to establish current practice and adequacy of reporting and conduct of NS of quantitative data in public health systematic reviews.

## 2 Methods

To assess reporting and conduct of NS, we identified a random sample of recent public health systematic reviews and systematically assessed the adequacy of reporting and conduct by benchmarking against available published guidance. The methods of this review are described below; further details are available in the review protocol [8].

To establish existing guidance on NS, we consulted publications, textbooks, and methods articles; these are outlined in Box 1, along with the key elements of NS from the most comprehensive guidance provided by Popay et al. [9] For the purposes of this work, we used the definition of NS as proposed by Popay et al. in the UK's Economic and Social Research Council (ESRC) guidance:

“Narrative synthesis refers to an approach to the systematic review and synthesis of findings from multiple studies that relies primarily on the use of words and text to summarise and explain the findings of the synthesis. Whilst narrative synthesis can involve the manipulation of statistical data, the defining characteristic is that it adopts a textual approach to the process of synthesis to ‘tell the story’ of the findings from the included studies”.([9], page 5)

Box 1 **Overview of ESRC guidance on narrative synthesis [9] and additional key sources consulted to establish best practice in narrative synthesis**

The most comprehensive guidance on the conduct and reporting of NS was published in 2006 [9], commonly known as the “ESRC guidance on NS”. The general elements of narrative synthesis set out by Popay et al. [9] (page 12–16):

1. Developing a theoretical model of how the interventions work, why, and for whom.
2. Developing a preliminary synthesis: develop an initial description of the results of included studies. Tools and techniques suggested: textual descriptions of studies, groupings and clusters, tabulation, transforming data into a common rubric, vote counting, translating data thematic analysis, content analysis.
3. Exploring relationships in the data: examine emerging patterns in data to identify any explanations for differences in direction or size of effect across included studies. Tools and techniques suggested: graphs, frequency distributions, funnel plots, forest plots, moderator variables and sub group analysis, idea webbing and conceptual mapping, translation reciprocal and refutational, qualitative case descriptions, investigator/methodological triangulation, conceptual triangulation.
4. Assessing the robustness of the synthesis product: trustworthiness of the synthesis, incorporating the methodological quality of the included studies and the methods used in the synthesis. Tools and techniques suggested: weight of evidence, best evidence synthesis, use of validity assessment, reflecting critically on the synthesis process, checking the synthesis with authors of primary studies.

Additional sources consulted to develop data extraction tool:

5. An introduction to systematic reviews [10].
6. Systematic reviews in the social sciences: a practical guide [11].
7. Synthesizing qualitative and quantitative health evidence: a guide to methods [12].
8. Guidelines for systematic reviews of health promotion and public health interventions [13].
9. Cochrane handbook for systematic reviews of interventions [5].
10. WHO Handbook for guideline development [14].

### 2.1 Search strategy, inclusion criteria, and review selection

We obtained a download of systematic reviews, from the McMaster Health Evidence database (http://www.healthevidence.org/), which were published between January 2010 and October 2015 inclusive. The Health Evidence database contains systematic reviews relevant to public health, which meet each of the following criteria: address questions related to promotion, protection, or prevention in public health or health; include participants from developed countries; examine an intervention/programme/service/policy; include evidence on outcomes; and describe a search strategy (see http://www.healthevidence.org/our-appraisal-tools.aspx). The Health Evidence database uses a validated search filter, which has high sensitivity, specificity, and precision for retrieving systematic reviews of public health interventions [15]. In addition to the database inclusion criteria, we specified that reviews had to be systematic and contain synthesis; we excluded expert reviews, overviews, empty reviews, and reviews with no synthesis.

Using the Microsoft Excel random number function, a 20% random sample was selected from the full Health-Evidence database download. The Excel random number function was used to allocate a number to each database entry (the results of the Health Evidence database search), and numbers were sorted lowest to highest. The first 20% of the random numbers were used to identify and include the corresponding Health Evidence reviews. This sample of reviews was screened (by M.C., H.T., A.S., S.V.K.) to identify reviews using NS of quantitative data for their primary outcome. If the review did not state a primary outcome, we identified the “primary outcome” of interest by the review question(s). A further 30% subsample of reviews, which used NS as the primary method of synthesis was randomly selected for more detailed data extraction and analysis.

### 2.2 Data extraction

The data extraction form was designed to reflect key elements of good practice in the conduct and reporting of NS of quantitative data. Key sources on the conduct of NS of quantitative data [10], [11], [12], [13], [14], [16] informed the design of the data extraction form (See Box 1). Three members of the research team (M.C., H.T. and S.V.K.) read the key sources independently and prepared a list of items or components that were common in the key sources. The lists were then collated to prepare items for inclusion in the draft data extraction form, which were then finalized in discussion with all authors (online Supporting Information file, Appendix Table S1). There was little variation in recommended practice for NS across the identified sources. The ESRC guidance provided the most comprehensive explanation and the other sources appeared to draw heavily on this guidance [9]. The data extraction form, therefore, largely reflects the core components recommended in the ESRC guidance. Five main aspects of NS were identified and covered by the data extraction exercise, namely:

• Reporting of NS methods
• Use of theory (i.e., articulation of how the intervention is expected to work)
• Management and investigation of heterogeneity across studies
• Transparency of data presentation and links to narrative
• Assessment of robustness of the synthesis (i.e., reflection of the synthesis methods used to assess the strength of the evidence from the included studies)

Two reviewers (M.C. and H.T.) independently piloted the data extraction form. All members of the project team conducted data extraction on a selection of the same five reviews until assessments were consistent across each member of the research team (M.C., H.T., S.V.K., and A.S.). The data were entered directly into a Microsoft Excel database. Health Evidence quality assessment ratings of the reviews were gathered after the data extraction exercise was complete.

### 2.3 Summarizing the data

The extracted data were tabulated to reflect the five main aspects of NS (see above) and are described narratively, with frequencies and descriptive data. Text was extracted to illustrate the reporting of NS methods.

## 3 Results

**Fig. 1:**
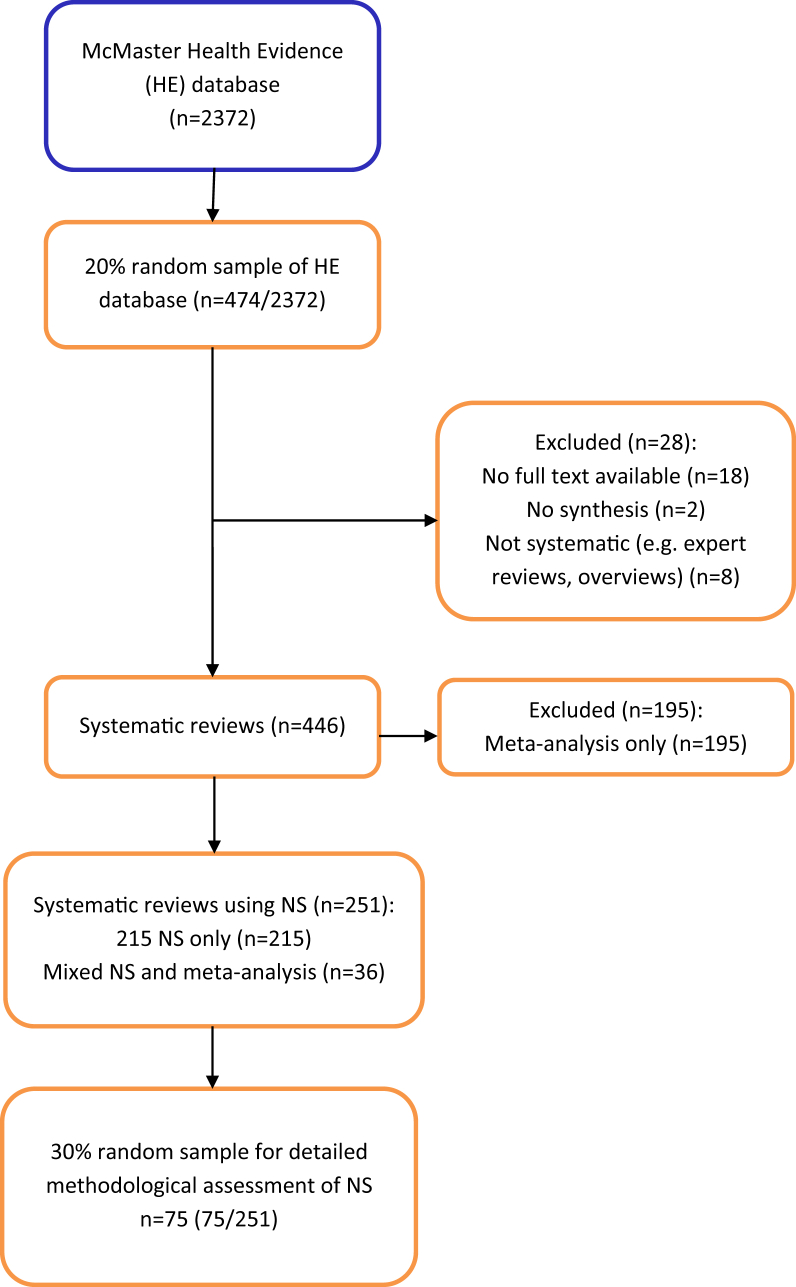
Review selection flow chart.

A total of 2,372 systematic reviews of public health interventions published between January 2010 and October 2015 were available from The McMaster Health Evidence database (see Fig. 1). From the initial 20% (*n* = 474/2,372) random sample of reviews, 28 (6%) were excluded as they did not fit our inclusion criteria: not systematic review (expert review/overview) (*n* = 8) or were empty reviews (contained no studies) (*n* = 2). We were unable to retrieve the full text of 18 further reviews. Of the 446 reviews included, 251 (56%) synthesized the data for the primary outcome narratively; of these, 215 (48%) used NS exclusively, and 36 (8%) used a combination of NS and meta-analysis for primary outcome data (i.e., some data were included in the meta-analysis, with other data reported and discussed in the narrative text). The remaining reviews (44%, *n* = 195) used meta-analysis to synthesize the primary outcome data.

### 3.1 Included reviews

All of the included reviews were published in international peer review journals. For a list of the included reviews, see Appendix Table S2. A list of results of extracted items reported in the text of this article is provided in Appendix Table S3. The McMaster Health Evidence database provides a quality assessment of each included review; this is based on a 10-item quality assessment tool that covers all aspects of the systematic review process. The assessment incorporates clarity of review question, appropriate search strategy, and risk of bias assessment, and two items assessing aspects of synthesis (“Was it appropriate to combine the findings of results across studies?”, “Were appropriate methods used for combining or comparing results across studies?”) (https://www.healthevidence.org/our-appraisal-tools.aspx). We randomly selected and analyzed the 75 reviews in our sample blind to the Health Evidence quality assessment scores and retrieved these scores after our data extraction exercise was complete. Of the reviews in our sample, 37% had a strong rating (score of 8 to 10/10), 60% moderate rating (score of 5 to 7/10), and 3% weak rating (score of 1 to 4/10). Therefore, we are confident that the majority of the sample reviews followed good practice; however, that assessment process did not fully examine the synthesis processes in the systematic reviews.

The following sections report on the detailed data extraction conducted on the 30% (*n* = 75/251) random sample of the reviews that synthesized data narratively.

### 3.2 Reporting of narrative synthesis methods

While 75 reviews synthesized data narratively, that is, using text only, a description of the methods used for NS was absent in 95% of the reviews (*n* = 71). Where methods were reported, the description was typically sparse, see examples in Box 2. Few review authors used the term “narrative synthesis” to describe their synthesis; 27% (*n* = 20/75) described their synthesis as “narrative” or “qualitative”, and justification for using NS was rarely provided (15%, *n* = 3/20). In around half (51%, *n* = 38/75) of the reviews using NS, the authors stated that they were unable to conduct a meta-analysis but provided no further details of how the data were synthesized (Table 1, items 1.1–1.3).

Box 2 **Examples of narrative synthesis description**

Examples of narrative synthesis description.

“A narrative synthesis was undertaken for each category of intervention to compare the effects of each on cervical screening uptake” Albrow R, Blomberg K, Kitchener H, et al. *Acta Oncologica* 2014; 53:445–51.
“The heterogeneous nature of the literature meant that a largely narrative synthesis approach was employed (citation provided).” Abendstern M, Harrington V, Brand C, Tucker S, Wilberforce M, Challis D. *Aging Ment Health* 2012; 16:861–73.
“Because of heterogeneity in outcomes and outcome assessment methodology, meta-analysis was not undertaken. Results are presented in narrative form.” Golley RK, Hendrie GA, Slater A, Corsini *N*. *Obesity Rev* 2011; 12:114–30.
“Results are presented as a narrative synthesis. Equity effect was summarised [citation provided].” Gallo MF, Nanda K, Grimes DA, Lopez LM, Schulz KF. *Cochrane Database Syst Rev* 2013; 2013:Art. No.: CD003989.
“Due to variability in participant and intervention characteristics, assessment tools used to diagnose frailty, and outcome measures used across studies, a meta-analysis could not be satisfactorily performed. Meta-analysis should only be considered when a group of studies have sufficient homogeneity between participants, interventions, and outcomes to provide a meaningful summary. In accordance with the Cochrane library if there is substantial clinical diversity a qualitative approach combining studies is appropriate.” Theou O, Stathokostas L, Roland KP, et al. *J Aging Res* 2011; 2011: Art. no: 569194.
For mixed meta-analysis and narrative synthesis: “Two studies that were conducted in children were not included in the meta-analyses and are reported separately.” Balk EM, Earley A, Raman G, Avendano EA, Pittas AG, Remmington PL. *Ann Intern Med* 2015: 437–51.

**Table 1:** Reporting and conduct of narrative synthesis

Review features	Reviews that synthesized data narratively (*n* = 75)	
1 Reporting narrative synthesis methods and use of theory		
1.1 Method of narrative synthesis described	Yes	5% (*n* = 4)
	State did NS, no description	16% (*n* = 12)
	No mention of NS	79% (*n* = 59)
1.2 Do authors state they will conduct narrative synthesis?	Yes	27% (*n* = 20)
	No	73% (*n* = 55)
1.3 What justification is given for using narrative synthesis?	Cannot conduct meta-analysis	51% (*n* = 38)
	NS most appropriate method	4% (*n* = 3)
	Providing summary of data	3% (*n* = 2)
	No justification provided	5% (*n* = 4)
	N/A (did not say would do NS)	37%(*n* = 28)
1.4 Theory/rationale for how the intervention(s) of interest is expected to work (before synthesis)	Explicit	47% (*n* = 35)
	Implicit	43% (*n* = 32)
	None	10% (*n* = 8)
2 Management and investigation of heterogeneity across studies		
2.1 Were data/studies split into subgroups for presentation of synthesis?	Yes	80% (*n* = 60)
	No	20% (*n* = 15)
2.2 If data/studies not split into subgroups, was there justification for this?	Yes	0% (*n* = 0)
	No	20% (*n* = 15)
	N/A (data split into subgroups)	80% (*n* = 60)
2.3 If studies were grouped/split, how were the studies grouped?	(multiple groupings in some reviews)	
	Study design	(*n* = 13)
	Risk of bias	(*n* = 5)
	Intervention	(*n* = 36)
	Population	(*n* = 9)
	Context (country, location/setting)	(*n* = 6)
	Outcome	(*n* = 26)
	Other	(*n* = 6)
	(Other = whether replication studies available [1], mechanisms [1], theoretical basis [3], comparisons [1])	
2. 4 Did review authors identify heterogeneity in the direction of the primary outcome?	Yes	19% (*n* = 14)
	No	60% (*n* = 46)
	Unclear	21% (*n* = 15)
2.5 If the authors reported heterogeneity in direction of primary outcome, was there any attempt to explain this?	To a large extent	2% (*n* = 1)
	To some extent	13% (*n* = 10)
	No	9% (*n* = 7)
	N/A	75% (*n* = 56)
	(on some occasions we commented on an “unclear whether heterogeneity identified” item)	
3 Transparency of data presentation and links to narrative		
3.1 Did presentation of data facilitate clear links between the text and the data for the reader?	Yes	57% (*n* = 43)
	Partially	32% (*n* = 24)
	No	5% (*n* = 4)
	No data presented in a table	5% (*n* = 4)
3.2 The summary of characteristics table(s) provide details of:	Study design	95% (*n* = 71)
	Risk of bias	52% (*n* = 39)
	Intervention	95% (*n* = 71)
	Population	88% (*n* = 66)
	Outcome	88% (*n* = 66)
	Context (country, location/setting)	65% (*n* = 49)
	Other	47% (*n* = 35)
	(Other includes: sampling strategy, theory, follow-up time, details of study control groups, brief results)	
3.3 In the conclusion, are the key findings clearly referring back to evidence in results (text or table/figure)?	Yes	60% (*n* = 45)
	To some extent	33% (*n* = 25)
	Unclear	7% (*n* = 5)
4 Robustness of synthesis		
4.1 Authors' reflections on limitations of synthesis	Free text, broadly coded:	
	Inclusion criteria	35% (*n* = 26)
	Heterogeneity	21% (*n* = 16)
	(study characteristics, outcomes, and analysis)	
	Generalizability of review findings	4% (*n* = 3)
	Analysis	11% (*n* = 8)
	(alternative analysis/coding possible lack of meta-analysis)	
	No mention of limitations of synthesis	36% (*n* = 27)
4.2 Authors' reflections on limitations of evidence	Free text, broadly coded:	
	Inadequate study quality	32% (*n* = 24)
	Lack of high-quality evidence	13% (*n* = 10)
	Relevant/available studies	19% (*n* = 14)
	Lack of intervention details	19% (*n* = 14)
	Heterogeneity of measurement outcomes	5% (*n* = 4)
	No mention of limitations of evidence	12% (*n* = 9)

Ten reviews (13%) reported the type of synthesis approach that was followed or referred to specific guidance or methods texts: ESRC guidance (*n* = 2) [9]; National Institute for Health and Care Excellence guidelines (*n* = 1) [16]; the Cochrane handbook (*n* = 2) [5]; thematic synthesis (*n* = 1) [17]; integrative review (*n* = 1) [18]; “formative” review (*n* = 1); “freeplane” (*n* = 1); and vote counting (*n* = 1).

### 3.3 Use of theory

Nearly all (90%, *n* = 67) of reviews reported how the intervention was expected to work or impact on the primary outcome. Around half of the reviews (47%, *n* = 35) did this explicitly, with two including a visual diagram to illustrate the mechanisms of action. A further 10% (*n* = 8) did not report any theory of change (Table 1, item 1.4).

### 3.4 Management and investigation of heterogeneity across studies

Diversity of study characteristics was dealt with in most (80%, *n* = 60) reviews by creating categories, usually by intervention, outcomes, or study design before conducting and presenting the synthesis (Table 1, item 2.1, 2.3). Two reviews (3%) reported conducting preliminary synthesis, a component of NS recommended in the ESRC guidance on NS [9].

A small number of reviews (19%, *n* = 14) reported heterogeneity in the direction of effect in the reported outcomes (positive, negative, or null effect, for the primary outcome) (Table 1, item 2.4). The lack of protocols for most reviews prevented recording whether investigation of heterogeneity was prespecified. This study was not assessing the appropriateness of the investigation of heterogeneity. This would require expertise in the topic of investigation for all the reviews, which our project team did not have. Rather, we describe how investigation of heterogeneity was conducted. Only one review investigated heterogeneity in the direction of effect; specifically, the authors explored differences in intervention components (treatment regimens) across studies and provided an explanation for the heterogeneity. Ten reviews provided hypothetical explanations for the variance in reported effect directions and three reviews did not offer any explanation. Hypothesized explanations for heterogeneity focused on differences in the characteristics or outcome measures of interventions, or the risk of bias of included studies. In one review (2%), the authors linked their hypothesized explanation of heterogeneity in reported effects to a prespecified theory, suggesting that intervention adherence influenced the outcome.

### 3.5 Transparency of data presentation and links to narrative

Tables presenting outcome data were provided in 85% (*n* = 64) of reviews, either alongside the text or as an online appendix. Although 54% (*n* = 40) of the reviews made the full data extraction available, either in the article (43%, *n* = 32) or online (11%, *n* = 8), the remaining 47% (*n* = 35) of reviews did not provide access to all the data incorporated into the synthesis. In 15% (*n* = 11) of reviews, not all the included studies were referred to in the narrative, leading to uncertainty as to whether the data from these studies had been included.

Using information about the type, detail, and clarity (including grouping) of reporting of data in each review, we assessed transparency; 57% (*n* = 43) of reviews were assessed as promoting transparent links between the data and the text. A summary table presenting key characteristics of included studies was included in 97% (*n* = 73) of reviews, providing information about study design, intervention, population, and outcomes (Table 1, item 3.1, 3.2).

We also assessed the extent to which review conclusions were linked to the included data, based on how clearly the conclusions referred to the reported results. We judged this to be clear, (i.e., the key findings in the conclusion clearly referred back to the text or visual evidence in the results), to a large extent or to some extent for most reviews (*n* = 45 and *n* = 25, respectively); however, in 7% (*n* = 5) of reviews, there was no clear link between the conclusions and the evidence referred to in the synthesis.

### 3.6 Assessment of the robustness of the synthesis

When considering the strengths and limitations of the evidence, review authors were more likely to reflect on the limitations of the primary studies included in the review (88%, *n* = 66), rather than limitations of the synthesis they had conducted (64%, *n* = 48). Limitations referred to risk of bias in included studies, relevance and reporting of study and intervention details, and heterogeneity of outcome measurements (Table 1, item 4.1). Where limitations of the synthesis were reported, these included search and inclusion criteria (e.g., search limited to published articles, only English language text included), heterogeneity of study characteristics, outcomes, and generalizability of the review findings to other settings or populations (Table 1 item 4.2).

Each assessor provided an overall subjective assessment of the level of trust in the results of each synthesis; 44% (*n* = 33) were considered to be trusted “to a large extent”, 44% (*n* = 33) “to some extent”, and “did not trust the synthesis” in 12% (*n* = 9) of reviews assessed. See Appendix Table S4 for comparison of the project team's level of trust of review syntheses with the Health Evidence quality rating.

## 4 Discussion

Narrative synthesis is more commonly used than meta-analysis for synthesizing quantitative data in systematic reviews of public health interventions. Despite its popularity, our detailed assessment shows that reporting of NS methods is almost totally absent, and the transparency of how NS is conducted is variable and currently inadequate. In 95% of reviews relying on NS for their primary outcome, all from international peer review journals, the methods used were not described. Although the majority of reviews did incorporate some core components of good practice (describing the rationale for the intervention, transparently relating tabulated data to the text in the results, and reflecting on the robustness of the synthesis), fewer than 30% of the reviews adopted each of these components. Our findings support previous criticism of NS as being opaque, particularly in relation to interpreting the evidence and being susceptible to selective reporting. This potential for bias is important and threatens the value of systematic reviews that use NS. In public health, where NS is commonly used, these are important issues undermining the role of these key resources as tools to support evidence-informed decision-making in public health.

The findings of our work are based on a representative sample of reviews from the Health Evidence database: a comprehensive source of systematic reviews of public health interventions [15]. Limitations of our study include the lack of a gold standard with which to compare reporting of NS. We used single assessors for data extraction; however, this was only after good agreement in the data extraction was achieved between independent assessors. Our sample of reviews allows an overall assessment of current practice within public health reviews, but we are aware that the sample is too small to allow robust comparison of reporting and conduct in reviews from different disciplines or different health topics. Despite the focus on public health, the findings are likely to be relevant to the wider field of evidence synthesis, regardless of topic. Indeed, we suspect that the conduct of NS may be poorer in other topic areas where there is less familiarity with NS as a method. NS will continue to be a necessary method of synthesis due to the complex nature of many interventions and the need to support evidence-informed decision-making [19].

The limited reference to available guidance on NS and the near absence of reporting of NS methods suggests that there is a general lack of familiarity with NS as a method among review authors. Furthermore, the lack of justification for using NS beyond statements such as “it was not possible to conduct meta-analysis” suggests that review authors may not consider NS to be a discrete method of synthesis. This is supported by our own informal discussions with experienced review authors who have expressed uneasiness around how to conduct and assess NS, yet acknowledge that NS is an important and essential method for reviews with high levels of heterogeneity and where diverse types of evidence are included.

Despite its frequent use, development of NS methods has been scant. This is in contrast to work to promote rigor in statistical synthesis or meta-analysis, [5] as well as more recent work to improve synthesis of qualitative data [17], [20], [21]. Similarly, reporting guidelines for meta-analysis (PRISMA) [22], meta-ethnography (EMERGE) [23], and synthesis of qualitative data (ENTREQ) [24] are widely available, yet relatively little has been written on how to promote transparency in the conduct and reporting of NS. This further supports the notion that NS of quantitative data is not widely recognized as a discrete synthesis method.

Increasingly, systematic reviews need to address questions about complex interventions and go beyond straightforward questions of effectiveness [3], [4], [19], [25], [26], [27], [28]. This issue goes beyond public health; the Cochrane 2020 strategy points to a move toward incorporating more diverse sources of evidence and addressing complex health decision-making questions [29]. NS is well placed to support these types of reviews, not only as an alternative when meta-analysis is contraindicated but also as an important synthesis tool in its own right. It offers a method for exploring and understanding the underlying arguments and justification of claims made in the included studies of a review [28]. NS enables reviewers to incorporate diversity in study designs, participants, interventions, or outcomes.

NS is likely to remain an important method for bringing together heterogeneous evidence. The work reported here shows that current practice in the conduct and in particular, the reporting of NS, is not consistent with the standards of transparency expected from rigorous and reliable systematic reviews. There is a need to provide support to those conducting NS and those attempting to assess the reliability of NS of quantitative data. NS is used in Cochrane reviews, perhaps more often than presumed. We estimated at least 20% of recent Cochrane reviews that used NS to synthesize outcome data [30]. We intend to contribute to the improved use of NS with the Improving the Conduct and reporting Of Narrative Synthesis of Quantitative data (ICONS-Quant) project, supported by the Cochrane Strategic Methods Fund, which aims to produce guidance and reporting guidelines for authors conducting NS of quantitative data (http://www.equator-network.org/library/reporting-guidelines-under-development/#74). Improved guidance has been linked to improved reporting of research [31], without which it is difficult for decision-makers to make use of research findings in the real world [32].

## 5 Conclusion

Narrative synthesis is a valuable method for synthesizing quantitative data where meta-analysis is not appropriate. Although NS of quantitative data is widely used, it is poorly reported and transparency is often lacking, threatening the credibility and value of many systematic reviews. The poor reporting suggests a lack of familiarity with, and confidence about, how to implement best practice when conducting NS. Improved guidance on the conduct and reporting of NS of quantitative data is required to support authors and ensure reviews using NS can be reliably used by decision-makers.

## Supplementary data

Data Profile

Transparency in Narrative Synthesis Supplementary File
