# Supplementary material for: Lack of transparency in reporting narrative synthesis of quantitative data: a methodological assessment of systematic reviews
Source: J Clin Epidemiol. 2019 Jan;105:1–9. doi: 10.1016/j.jclinepi.2018.08.019 (PMC6327109; doi:10.1016/j.jclinepi.2018.08.019)
Supplement: Transparency in Narrative Synthesis Supplementary File [file mmc2.docx]

**Web-only Appendix**

- **Table S1:** Data extraction template
- **Table S2:** List of 75 included systematic reviews
- **Table S3:** Further data extraction items
- **Table S4:** Comparison of project team’s level of trust with Health Evidence quality rating

**Table S1: Data extraction template**

| **Item number** | **Label** | | **Explanation** |
| --- | --- | --- | --- |
| 0.1 Pre-question | Does the review use narrative synthesis? | | *Dropdown list options:*  Yes (there is some form of synthesising /combining the data)  No (there is merely an individual summary of each included study) |
|  |  | | Column for providing comments for 0.1 above |
| **Review characteristics** | | | |
| 1.1 | Author | | Surname of first author |
| 1.2 | Year | | Year paper published |
| 1.3 | Endnote reference | | Endnote record number |
| 1.4 | Journal | | Title of journal |
| 1.5 | Question | | State the research question |
| 1.6 | Narrative synthesis used | | NS only / mixed NS and meta-analysis |
| 1.7 | Intervention | | Intervention under review |
| 1.8 | Outcomes | | Primary outcome(s) of interest in systematic review (state what these are, or ‘outcomes of the included studies’ |
| 1.9 | Population | | *(Free text)*  Briefly state population of interest |
| 1.10 | Context | | *(Free text)*  If the review notes any context of importance in the methods, state what this is, or state ‘none reported’. |
| 1.11 | Study designs of included studies **planned** | | *(Free text)*  State what the review authors reported in the methods section of the review would be included quantitative study designs  *Dropdown list options:*  RCT  RCT & NRS  NRS  RCT & other- qual  RCT & NRS & other-qual  NRS & other-qual  Other (add details in next comments column) |
|  | Comments | | Column for providing details of 'other' in 1.11 above |
| 1.12 | Study designs **actually included** in the review | | State what the review authors reported in the **results/findings** section were the included quantitative study designs  *Dropdown list options:*  RCT  RCT & NRS  NRS  RCT & other- qual  RCT & NRS & other-qual  NRS & other-qual  Other (add details in next comments column) |
|  | Comments | | Column for providing details of 'other' in 1.12 above |
| 1.13 | Did the paper refer to a protocol for the systematic review? | | yes /no |
|  | Comments | | Column for any comments/notes for ‘Review characteristics’ section |
| **Theory** | | | |
| 2.1 | Does the review set out the rationale for how the intervention(s) of interest is expected to work (prior to the review analysis)? | | *Dropdown list options:*  **Explicit =** clear explanation of the theory of change  **Implicit =** there is vague, indirect mention of how intervention may be linked to outcomes but does not state theory.  **Not stated** = no reasoning given for how intervention of interest is expected to work  **N/A** = main focus not on effectiveness of interventions |
| 2.2 | Is there a logic model/schematic explaining the theory? | | *Dropdown list options:*  visual included  no visual  N/A (no theory) |
|  | Comments | | Column for any comments/notes for ‘Theory’ section |
| **Data presentation** | | | |
| 3.1 | Was there a summary table presenting key characteristics of included studies? | | *Dropdown list options:*  Yes  No |
| 3.2 | Did the summary table(s) give information about:  study design  risk of bias  intervention  population  context  outcome  other (state what this is in following column) | | (Separate question for each characteristic)  *Dropdown list options:*  Yes  No  N/A |
|  | Comments | | Column for “other” details, or comments for 3.2 |
| 3.3 | Did the review have a table presenting the data of the studies? | | *Dropdown list options:*  Yes  No |
| 3.4 | Did the presentation of included data reflect the groupings used in the synthesis? | | *Dropdown list options*:  Yes, arranged by characteristics that are used to report synthesis (e.g. table groups all RCT studies together and synthesis is by study design. Or: only one group in synthesis)  Unclear  No, studies different grouping for summary of key characteristics and synthesis  N/A (no grouping in synthesis) |
|  | Comments | | Excel column for comments details of “other” above in 3.4) |
| 3.5 | Did presentation of data facilitate clear links between the text and the data for the reader? | | *Dropdown list options:*  Yes  Partially  No |
| 3.6 | Were all the included studies accounted for in the TEXT reporting of the synthesis of the primary outcome | | *Dropdown list options:*  Yes  No |
| 3.7 | Was the full data extraction made available to the reader? [list] | | *Dropdown list options:*  ‘full data extraction published with paper’  ‘full data extraction in online appendix’  ‘full data extraction available from author’  ‘limited data provided in paper only’  Other (add details in next comments column) |
| 3.8 | Did authors report assessing the quality of the included studies? | | *Dropdown list options:*  Yes  No |
|  | Comments | | Excel column for comments details of “other” above in 3.8) |
|  | Comments | | Column for any comments/notes for ‘Data presentation’ section |
| **Synthesis methods in the review** | | | |
| 4.1 | Synthesis methods reported in the review | Cut and paste synthesis methods from review | |
| 4.2 | Do authors state they will conduct narrative synthesis? | *Dropdown list options:*  Yes  No | |
| 4.3 | What justification is given for using narrative synthesis? | *Dropdown list options:*  Not able to do a meta-analysis or too much heterogeneity  NS is best method for data  Providing summary of data  No justification synthesis/summary  N/A (did not say would do NS)  Other (provide details in following column) | |
|  | Comments | Excel column for comments / details of “other” above in 4.3 | |
| 4.4 | Is the method of narrative synthesis described? | *Dropdown list options:*  **Yes** (they outline of what do to synthesise, e.g. plan to look at particular aspects, or will follow a method or guidance, etc.)  **Just state will do narrative synthesis** (just simply report will do a narrative synthesis/summary)  **No** – no mention of synthesis methods | |
| 4.5 | Any stated type of synthesis approach? | *Dropdown list options:*  Yes  No | |
|  | If yes synthesis approach stated in 4.5, provide details | *(Free text)* | |
| 4.6 | Was there any reference to preliminary synthesis? | *Dropdown list options:*  Yes  No | |
|  | Comments | Column for any comments/notes for ‘Synthesis methods’ section | |
| **Management of heterogeneity** | | | |
| 5.1 | Were data/studies split into sub-groups for presentation of synthesis? | | *Dropdown list options:*  Yes  No |
| 5.2 | If data/studies not split into sub-groups, was there justification for this? | | *Dropdown list options:*  N/A (data/studies split into groups)  Yes (provide details in following comments column)  No |
|  |  | | Excel column for details of “yes” above in 5.2 |
| 5.3 | If studies were grouped/split, how were the studies grouped?:  study design  risk of bias  intervention  population  context  outcome  other (state what this is) | | (Separate question for each characteristic)  *Dropdown list options:*  Yes  No  N/A |
|  | Comments | | Excel column for comments / details of “other” above in 5.3 |
| 5.3 continued | Was there justification provided for grouping studies/data by:  study design  risk of bias  intervention  population  context  outcome  other (state what this is in following column) | | (Separate question for each characteristic)  *Dropdown list options:*  Yes  No  N/A |
|  | Comments | | Excel column for comments / details of “other” above in 5.4 |
| 5.4 | Did review authors identify heterogeneity in the direction of the **primary** outcome? | | *Dropdown list options:*  Yes  No  Unclear/difficult to tell |
| 5.5 | If the authors reported heterogeneity in direction of primary outcome, was there any attempt to explain this? | | *Dropdown list options:*  N/A (no heterogeneity reported/5.4 ‘unclear’)  To **some extent** (e.g. hypothesis based on study characteristics)  To a **large extent** (some form of further analysis to investigate heterogeneity in direction)  No |
| 5.6 | How was heterogeneity in reported findings investigated? (i.e. what factors were investigated) | | *(Free text)*  State what factors were investigated  Or N/A |
| 5.7 | If there was heterogeneity and this was investigated, did the investigation relate to a pre-specified theory of change? | | *Dropdown list options:*  Yes  No  Unclear  N/A Heter (no heterogeneity)  N/A Theory (no pre-specified theory) |
| 5.8 | Was heterogeneity in the size/direction of primary outcome(s) explained? | | *Dropdown list options:*  N/A (no heterogeneity)  Explained  Hypothesised explanation  Unexplained despite investigation  Not investigated |
|  | Comments | | Column for any comments/notes for ‘Management of heterogeneity’ section |
| 6.1 | In the conclusion, are the key findings clearly referring back to evidence in results (text or table/figure)? | | *Dropdown list options:*  Yes, key conclusion(s) clearly reflect data reported in results section of review  To some extent conclusion reflects data in results  Unclear how conclusions directly relate to results section of review |
|  | Comments | | Excel column for comments / details of “other” above in 7.1 |
| 6.2 | Authors’ reflections on limitations of synthesis | | *(Free text)*  State briefly what limitations of synthesis the review authors reported |
| 6.3 | Authors’ reflections on limitations of the evidence included in the synthesis | | *(Free text)*  State briefly what limitations of evidence included in the synthesis the review authors reported |
|  | Comments | | Column for any comments/notes for ‘Robustness of synthesis’ section |
| 6.4 | Would you trust the results of the synthesis? | | In your opinion, how much do you trust the results of the **narrative synthesis**  *Dropdown list options:*  To a large extent  To some extent  No |

**Table S2: List of references of sample of systematic reviews**

| 1. Aantjes CJ, Ramerman L, Bunders JF. A systematic review of the literature on self-management interventions and discussion of their potential relevance for people living with HIV in sub-Saharan Africa. *Patient Educ Couns* 2014;**95**(2):185-200.  2. Aas RW, Tuntland H, Holte KA, Re C, Lund T, Marklund S, et al. Workplace interventions for neck pain in workers. *Cochrane Database Syst Rev* 2011; **4**: CD008160.  3. Abendstern M, Harrington V, Brand C, Tucker S, Wilberforce M, Challis D. Variations in structures, processes and outcomes of community mental health teams for older people: A systematic review of the literature. *Aging Mental Health* 2012;**16**(7):861-73.  4. Albrow R, Blomberg K, Kitchener H, Brabin L, Patnick J, Tishelman C, et al. Interventions to improve cervical cancer screening uptake amongst young women: A systematic review. *Acta Oncol* 2014;**53**(4):445-51.  5. Balk EM, Earley A, Raman G, Avendano EA, Pittas AG, Remmington PL. Combined diet and physical activity promotion programs to prevent type 2 diabetes among persons at increased risk: A systematic review for the community preventive services task force. *Ann Intern Med* 2015;**163**(6):437-51.  6. Barte H, Horvath TH, Rutherford GW. Yellow fever vaccine for patients with HIV infection. Cochrane Database of Systematic Reviews. 2014;2014(1):CD010929.  7. Bhattarai N, Prevost AT, Wright AJ, Charlton J, Rudisill C, Gulliford MC. Effectiveness of interventions to promote healthy diet in primary care: Systematic review and meta-analysis of randomised controlled trials. *BMC Public Health* 2013;**13**:1203.  8. Bleakley CMC, McCormack B. Gaming for health: A systematic review of the physical and cognitive effects of interactive computer games in older adults. *J Appl Gerontol* 2015;**34**(3):NP166-NP89.  9. Brown T, Platt S, Amos A. Equity impact of population-level interventions and policies to reduce smoking in adults: A systematic review. *Drug Alcohol Depend* 2014;**138**:7-16.  10. Burnhams NH, Musekiwa A, Parry C, London L. A systematic review of evidence-based workplace prevention programmes that address substance abuse and HIV risk behaviours. *African Journal of Drug and Alcohol Studies* 2013;**12**(1):2013.  11. Charach A, Carson P, Fox S, Ali MU, Beckett J, Lim CG. Interventions for preschool children at high risk for ADHD: A comparative effectiveness review. *Pediatrics* 2013;**131**(5):e1584-e604.  12. Cheney G, Schlosser A, Nash P, Glover L. Targeted group-based interventions in schools to promote emotional well-being: A systematic review. *Clin Child Psychol* Psychiatry 2013;**19**(3):412-38..  13. Clegg A, Siddiqi N, Heaven A, Young J, Holt R. Interventions for preventing delirium in older people in institutional long-term care. *Cochrane Database Syst Rev* 2014;**1**:CD009537.  14. Cusimano MD, Nassiri F, Chang Y. The effectiveness of interventions to reduce neurological injuries in rugby union: A systematic review. *Neurosurgery* 2010;**67**(5):1404-18.  15. De Cesaro BC, Gurgel LG, Nunes GP, Reppold CT. Child language interventions in public health: A systematic literature review. *Codas* 2013;**25**(6):588-94.  16. Denno DM, Chandra-Mouli V, Osman M. Reaching youth with out-of-facility HIV and reproductive health services: a systematic review. *J Adolesc Health* 2012;**51**(2):106-21.  17. Downs SM, Thow AM, Leeder SR. The effectiveness of policies for reducing dietary trans fat: A systematic review of the evidence. *Bull World Health Organ* 2013;**91**:262H-9H.  18. Dudley D, Okely A, Pearson P, Cotton W. A systematic review of the effectiveness of physical education and school sport interventions targeting physical activity, movement skills and enjoyment of physical activity. *Eur Phy Educ Rev* 2011;**17**:353-78.  19. Dunkley AJ, Charles K, Gray LJ, Camosso-Stefinovic J, Davies MJ, Khunti K. Effectiveness of interventions for reducing diabetes and cardiovascular disease risk in people with metabolic syndrome: Systematic review and mixed treatment comparison meta-analysis. *Diabetes Obes Metab* 2012;**14**(7):616-25.  20. Eisenberg CM, Sanchez-Romero LM, Rivera-Dommarco JA, Holub CK, Arredondo EM, Elder JP, et al. Interventions to increase physical activity and healthy eating among overweight and obese children in Mexico. *Salud Publica Mex* 2013;**55**(Suppl 3):441-6.  21. Everson H, Johnson M, Jones R, Woods HB, Goyder E, Payne N, et al. Community-based dietary and physical activity interventions in low socioeconomic groups in the UK: A mixed methods systematic review. *Prev Med* 2013;**56**(5):265-72.  22. Fleming-Dutra KE, Conklin L, Loo JD, Knoll MD, Park DE, Kirk J, et al. Systematic review of the effect of pneumococcal conjugate vaccine dosing schedules on vaccine-type nasopharyngeal carriage. Pediatr *Infect Dis J* 2014;**33**(2):S152-S60.  23. Gallo MF, Nanda K, Grimes DA, Lopez LM, Schulz KF. 20 mcg versus >20 mcg Estrogen combined oral contraceptives for contraception. *Cochrane Database Syst Rev* 2013; **8**: CD003989.  24. Gillies M, Palmateer N, Hutchinson S, Ahmed S, Taylor A, Goldberg D. The provision of non-needle/syringe drug injecting paraphernalia in the primary prevention of HCV among IDU: A systematic review. *BMC Public Health* 2010;**10**:721.  25. Golley RK, Hendrie GA, Slater A, Corsini N. Interventions that involve parents to improve children's weight-related nutrition intake and activity patterns - what nutrition and activity targets and behaviour change techniques are associated with intervention effectiveness? *Obes Rev* 2011;**12**(2):114-30.  26. Gould DJ, Moralejo D, Drey N, Chudleigh JH. Interventions to improve hand hygiene compliance in patient care. *Cochrane Database of Systematic Reviews* 2010; **9**:CD005186.  27. Guillaumier A, Bonevski B, Paul C. Anti-tobacco mass media and socially disadvantaged groups: A systematic and methodological review. *Drug Alcohol Rev* 2012;**31**(5):698-708.  28. Hahn EJ. Smokefree legislation: A review of health and economic outcomes research. *Am J Prev Med* 2010;**39**(6 Suppl 1):S66-S76.  29. Halpern V, Lopez LM, Grimes DA, Stockton LL, Gallo MF. Strategies to improve adherence and acceptability of hormonal methods for contraception. *Cochrane Database of Systematic Reviews* 2013;**10**: CD004317.  30. Hartmann-Boyce J, Cahill K, Hatsukami D, Cornuz J. Nicotine vaccines for smoking cessation. *Cochrane Database of Systematic Reviews* 2012; 8: CD007072.  31. Hawkes S, Matin N, Broutet N, Low N. Effectiveness of interventions to improve screening for syphilis in pregnancy: A systematic review and meta-analysis. *Lancet Infect Dis* 2011;**1**(9):684-91.  32. Hedman E, Ljotsson B, Lindefors N. Cognitive behavior therapy via the Internet: A systematic review of applications, clinical efficacy and cost-effectiveness. *Expert Rev Pharmacoecon Outcomes Res* 2012;**12**(6):745-64.  33. Hemila H, Louhiala P. Vitamin C for preventing and treating pneumonia. *Cochrane Database Syst Rev* 2013; **8**:CD005532.  34. Heo HH, Braun K. Culturally tailored interventions of chronic disease targeting Korean Americans: A systematic review. *Ethn Health* 2014;**19**(1):64-85.  35. Hindin MJ, Bloem P, Ferguson J. Effective nonvaccine interventions to be considered alongside human papilloma virus vaccine delivery. *J Adolesc Health* 2015;**56**(1):10-8.  36. Ho LC, Saunders KAL, Owen DJ, Ibrahim UNN, Bhattacharya S. Are antenatal weight management interventions effective in preventing pre-eclampsia? Systematic review of randomised control trials. *Pregnancy Hypertens* 2012;**2**(4):341-9.  37. Imhoff-Kunsch B, Briggs V, Goldenberg T, Ramakrishnan U. Effect of n-3 long-chain polyunsaturated fatty acid intake during pregnancy on maternal, infant, and child health outcomes: A systematic review. Paediatr *Perinat Epidemiol* 2012;**26**(Suppl 1):91-107.  38. Ingoldsby EM. Review of interventions to improve family engagement and retention in parent and child mental health programs. *J Child Fam Stud* 2010;**19**(5):629-45.  39. Jensen JD, Hartmann H, de MA, Schuit A, Brug J, Consortium E. Economic incentives and nutritional behavior of children in the school setting: A systematic review. *Nutr Rev* 2011;**69**(11):660-74.  40. Joo JY. Effectiveness of culturally tailored diabetes interventions for Asian immigrants to the United States: A systematic review. *Diabetes Education* 2014;**40**(5):605-15.  41. Keukenmeester R, Slot D, Putt M, Weijden G. The effect of sugar-free chewing gum on plaque and clinical parameters of gingival inflammation: A systematic review. *Int J Dent Hyg* 2013;**11**(1):2-14.  42. Kock R, Becker K, Cookson B, Van Gemert-Pijnen JE, Harbarth S, Kluytmans J, et al. Systematic literature analysis and review of targeted preventive measures to limit healthcare-associated infections by meticillin-resistant staphylococcus aureus. *Eurosurveillance* 2014;**19**(29):23-49.  43. Lopez LM, Otterness C, Chen M, Steiner M, Gallo MF. Behavioral interventions for improving condom use for dual protection. Cochrane Database of Systematic Reviews. 2013;2013(10):CD010662.  44. Maon S, Edirippulige S, Ware R, Batch J. The use of web-based interventions to prevent excessive weight gain. J Telemed Telecare. 2012;18(1):37-41.  45. Martin MS, Colman I, Simpson A, McKenzie K. Mental health screening tools in correctional institutions: A systematic review. *BMC Psychiatry* 2013;**13**:275.  46. Mateus AL, Otete HE, Beck CR, Dolan GP, Nguyen-Van-Tam JS. Effectiveness of travel restrictions in the rapid containment of human influenza: A systematic review. *Bull World Health Organ* 2014;**92**(12):868-80D.  47. Mathers M, Keyes M, Wright M. A review of the evidence on the effectiveness of children's vision screening. *Child Care Health Dev* 2010;**36**(6):756-80.  48. Milton B, Attree P, French B, Povall S, Whitehead M, Popay J. The impact of community engagement on health and social outcomes: A systematic review. *Community Dev J* 2012;**47**:316-34.  49. Moran AM, Coyle J, Pope R, Boxall D, Nancarrow SA, Young J. Supervision, support and mentoring interventions for health practitioners in rural and remote contexts: an integrative review and thematic synthesis of the literature to identify mechanisms for successful outcomes. *Human Resources for Health* 2014;**12**(10):1-30.  50. Neville CE, McKinley MC, Holmes VA, Spence D, Woodside JV. The effectiveness of weight management interventions in breastfeeding women: A systematic review and critical evaluation. *Birth Iss Perinat C* 2014;**41**(3):223-36.  51. Petering R, Wenzel S, Winestrobe H. Systematic review of current intimate partner violence prevention programs and applicability to homeless youth. *J Soc Social Work Res* 2014;**5**(1):107-35.  52. Poole MK, Seal DW, Taylor CA. A systematic review of universal campaigns targeting child physical abuse prevention. *Health Educ Res* 2014;**29**(3):388-432.  53. Rees K, Hartley L, Day C, Flowers N, Clarke A, Stranges S. Selenium supplementation for the primary prevention of cardiovascular disease. *Cochrane Database Syst Rev* 2013; **1**: CD009671.  54. Ruotsalainen JH, Verbeek JH, Marine A, Serra C. Preventing occupational stress in healthcare workers. *Cochrane Database Syst Rev* 2015;**4**: CD002892.  55. Ryan P, Schlidt A, Ryan C. The impact of osteoporosis prevention programs on calcium intake: A systematic review. *Osteoporos Int* 2013;**24**(6):1791-801.  56. Santa Maria D, Markham C, Bluethmann S, Mullen PD. Parent-based adolescent sexual health interventions and effect on communication outcomes: A systematic review and meta-analyses. *Perspect Sex Reprod Health* 2015;**47**(1):37-50.  57. Sheikh A, Hurwitz B, Nermatov U, van Schayck CP. House dust mite avoidance measures for perennial allergic rhinitis. *Cochrane Database Syst Rev* 2010; **7**: CD001563.  58. Shepherd J, Frampton GK, Harris P. Interventions for encouraging sexual lifestyles and behaviours intended to prevent cervical cancer. *Cochrane Database Syst Rev* 2011; 4: CD001035.  59. Siegfried NL, van der Merwe L, Brocklehurst P, Sint TT. Antiretrovirals for reducing the risk of mother-to-child transmission of HIV infection. *Cochrane Database Syst Rev* 2011; **7**:CD003510.  60. Steeves JA, Thompson DL, Bassett DR, Fitzhugh EC, Raynor HA. A review of different behavior modification strategies designed to reduce sedentary screen behaviors in children. *J Obes* 2012;**2012**:379215.  61. Szumilas M, Kutcher S. Post-suicide intervention programs: A systematic review. *C J Public Health* 2011;**102**(1):18-29.  62. Taylor C, Upton P, Upton D. Increasing primary school children's fruit and vegetable consumption: A review of the food dudes programme. *Health Educ* 2015;**115**(2):178-96.  63. Theou O, Stathokostas L, Roland KP, Jakobi JM, Patterson C, Vandervoort AA, et al. The effectiveness of exercise interventions for the management of frailty: A systematic review. *J Aging Res* 2011;**2011**:569194.  64. Thomas RE, Russell ML, Lorenzetti DL. Systematic review of interventions to increase influenza vaccination rates of those 60 years and older. *Vaccine* 2010;**28**(7):1684-701.  65. Thomas Y, Gray M, McGinty S. A systematic review of occupational therapy interventions with homeless people. *Occup Ther Health Care* 2011;**25**(1):38-53.  66. Tuckett A, Hodgkinson B, Hegney D, Paterson J, Kralik D. Effectiveness of educational interventions to raise men's awareness of bladder and bowel health. *Int J Evid Based Healthc* 2011;**9**(2):81-96.  67. Underdown A, Barlow J, Stewart-Brown S. Tactile stimulation in physically healthy infants: Results of a systematic review. *J Reprod Infant Psychol* 2010;**28**(1):11-29.  68. van der Meer RM, Willemsen MC, Smit F, Cuijpers P. Smoking cessation interventions for smokers with current or past depression. *Cochrane Database Syst Rev* 2013;8:CD006102.  69. Wang K, Brown K, Shen SY, Tucker J. Social network-based interventions to promote condom use: A systematic review. *AIDS Behav* 2011;**15**(7):1298-308.  70. Wolff R, Hommerich J, Riemsma R, Antes G, Lange S, Kleijnen J. Hearing screening in newborns: Systematic review of accuracy, effectiveness, and effects of interventions after screening. *Arch Dis Child* 2010;**95**(2):130-5.  71. Xiang X. A review of interventions for substance use among homeless youth. *Res Soc Work Pract* 2013;**23**(1):34-45.  72. Yassi A, Lockhart K, Sykes M, Buck B, Stime B, Spiegel JM. Effectiveness of joint health and safety committees: A realist review. *Am J Ind Med* 2013;**56**(4):424-38.  73. Young I, McDaid L. How acceptable are antiretrovirals for the prevention of sexually transmitted HIV?: A review of research on the acceptability of oral pre-exposure prophylaxis and treatment as prevention. *AIDS Behav* 2013;**18**(2):195-216.  74. Zaher E, Keogh K, Ratnapalan S. Effect of domestic violence training: Systematic review of randomized controlled trials. *Can Fam Physician* 2014;**60**(7):618-24.  75. Zhu L, Ho S, Wong TK. Effectiveness of health education programs on exercise behavior among patients with heart disease: A systematic review and meta-analysis. *J Evid Based Med* 2013;**6**(4):265-301. |
| --- |

**Table S3: Table of further data extraction items**

|  | **Reviews which synthesised data narratively (n=75)** |
| --- | --- |
| **Reporting narrative synthesis (NS) methods and use of theory** | |
| Any stated type of synthesis approach? | Yes 13% (n=10)  No 87% (n=65) |
| If yes synthesis approach stated in item above, provide details | Free text, collated:  following Cochrane handbook (n=2)  ESRC & CRD framework (n=2)  NICE guidelines (n=1)  integrative review (n=1)  “formative” review (n=1)  Freeplane - similar to Framework analysis (n=1)  Thomas and Harden (thematic synthesis) (n=1)  vote counting (n=1) |
| Was there any reference to preliminary synthesis? | Yes 3% (n=2)  No 97% (n=73) |
| **Management and investigation of heterogeneity across studies** | |
| Did the presentation of included data reflect the groupings used in the synthesis? | Yes 59% (n=44)  Unclear 4% (n=3)  No 20% (n=15)  N/A (no grouping) 12% (n=9)  No data presented in a table 5% (n=4) |
| **Transparency of data presentation and links to narrative** | |
| Was there a summary table presenting key characteristics of included studies? | Yes 97% (n=73)  No 3% (n=2) |
| Did the review have a table presenting the data of the studies? | Yes 85% (n=64)  No 15% (n=11) |
| Were all the included studies accounted for in the TEXT reporting of the synthesis of the primary outcome? | Yes 85% (n=64)  No 15% (n=11) |
| Was the full data extraction made available to the reader? | Full data extraction published with paper 43% n=32)  Full data extraction in online appendix 11% (n=8)  Full data extraction available from author 0% (n=0)  Limited data provided in paper only 44% (n=33)  Limited data available online 3% (n=2) |
| **Assessment of the robustness of the synthesis** |  |
| Did authors report assessing the quality of the included studies? | Yes 75% (n=56)  No 25% (n=19) |
| Would you trust the results of the synthesis? | To a large extent 44% (n=33)  To some extent 44% (n=33)  No 12% (n=9) |

**Table S4: Table of comparison of project team’s level of trust with the Health Evidence quality rating of the reviews.**

The data extraction was conducted blind to the quality rating assigned to each review by McMaster Health Evidence. The project team assessed their trust in the synthesis: to a large extent; to some extent; did not trust synthesis. Health Evidence Quality ratings of the review are scored one to ten, which Health Evidence categorise as high quality (score of 8 to 10/10), moderate (score of 5 to 7/10), and weak (score of 1 to 4/10). After the data extraction was complete, our assessors’ level of trust was compared to the Health Evidence categories. Table S4 shows the results of the comparison.

| **Level of matching** | **Amount of reviews** | **Explanation of matching between Health Evidence (HE) quality rating and project team trust of synthesis** |
| --- | --- | --- |
| Direct matches | 56% (n=42) | HE rated review ‘strong’ = project team trusted review 'to a large extent’  or  HE rated moderate = project team trusted review 'to some extent' |
| Matched rating within one level/point of each scale | 32% (n=24) | HE rated ‘strong’ = project team trusted 'to some extent’  or  HE rated moderate = Project team trusted 'to a large extent' |
| Ratings did not match | 12% (n=9) | All nine were reviews that the project team did not trust the synthesis and Health Evidence rated the review of moderate quality |
